# Comparison of CD63 Upregulation Induced by NSAIDs on Basophils and Monocytes in Patients with NSAID Hypersensitivity

**DOI:** 10.1155/2012/580873

**Published:** 2011-12-01

**Authors:** N. Abuaf, H. Rostane, J. Barbara, C. Toly-Ndour, H. Gaouar, P. Mathelier-Fusade, F. Leynadier, C. Francès, R. Girot

**Affiliations:** ^1^Service d'Hématologie et d'Immunologie Biologique, Hôpital Tenon (AP-HP), Université Pierre et Marie Curie, 4 Rue de la Chine, 75020 Paris, France; ^2^Service de Dermatologie et Allergologie, Hôpital Tenon (AP-HP), Université Pierre et Marie Curie, 4 Rue de la Chine, 75020 Paris, France

## Abstract

*Background*. An in vitro basophil activation test, based on the detection of CD63 upregulation induced by NSAIDs, has been described. Its clinical significance remains controversial. *Objectives*. In patients with a history of nonallergic NSAID hypersensitivity, stratified according to the severity of the symptoms, to assess with NSAIDs the predictive value of basophil (BAT) and monocyte (MAT) activation tests. *Patients/Methods*. Sixty patients who had NSAIDs-induced or exacerbated urticaria/angiooedema and 20 controls was included. After incubation with NSAIDs or acetaminophen, leukocytes were analysed for CD63 upregulation. *Results*. With aspirin, the sensitivity (37%) and specificity (90%) of BAT agree with already published results. In contrast, when patients had had cutaneous and visceral reactions, the frequency of positive BAT 14/22 (64%, *P* < 0.001) or MAT 10/22 (46%, *P* < 0.01) were increased. *Conclusions*. Positive tests were more frequent among patients having a severe hypersensitivity contrasting with the other patients who had results similar to controls.

## 1. Introduction

Nonsteroidal anti-inflammatory drugs (NSAIDs) are after antibiotics, the second most frequently suspected agents causing drug hypersensitivity. The prevalence of acetyl salicylic acid (ASA, aspirin) hypersensitivity ranges from 0.6% to 2.5% in the general population, from 4.3% to 11% in asthmatic patients [1], and from 20 to 40% in chronic idiopathic urticaria (CIU) [2]. It also occurs in subjects with no known underlying disease, otherwise normal when they abstain from taking NSAID.

Hypersensitivity may occur shortly, within 15 minutes or longer, up to 24 hours after NSAID intake. In general it develops within 1 to 4 hours [3]. Some patients might have life-threatening reactions, especially those with aspirin-exacerbated respiratory diseases (AERDs, Widal syndrome), which associate aspirin sensitivity, asthma, nasal polyposis, and airway remodelling [1].

In most patients the adverse reaction is nonallergic. Those with eicosanoid metabolism dysfunction or other alterations are prone to hypersensitivity when NSAIDs inhibit the enzyme cylooxygenase-1 (Cox-1) [3–14]. Selective NSAIDs strongly inhibit COX-2, but they are weak inhibitors of COX-1, so they are well tolerated in patients with NSAID-sensitive asthma or CIU [4, 5]. The concentration inhibiting efficiently COX-1 or COX-2 may differ as much as 3 logs between the strongest and weakest inhibitors (Table 1) [15–17]. Pharmacological profiles as well as hypersensitivity depend on their inhibitory activities.

The diagnosis of NSAID hypersensitivity is based on clinical histories and provocation challenges with aspirin or NSAIDs [19–21]. Skin test (ST) responses are typically negative except when there is a true allergy. Oral challenge tests rule out hypersensitivity in 50% of the patients [20] which suggests that clinical histories are not sufficient to diagnose true NSAID hypersensitivity. Due to the severe reactions that might occur in some patients, it was not desirable to use oral challenge systematically. There is a need for laboratory tests; hence flow cytometric determination of CD63 upregulation on basophils incubated with aspirin and other NSAIDs has been described. Sensitivity has been shown to be 43% with aspirin or diclofenac [7, 22, 23]. However, conflicting results have also been published about the specificity or the sensitivity of the test and the clinical significance [21, 24–26].

In ASA-induced urticaria or asthma, in addition to basophils, neutrophils or other leukocytes are also activated [8, 27]. The aim of the present study is in patients suffering from nonallergic NSAID hypersensitivity, stratified according to the severity of clinical symptoms, to compare the clinical performance of the basophil activation test (BAT) with the monocyte (MAT) activation test.

## 2. Patients and Methods

### 2.1. Patients

Sixty-five patients referred by the patients' physician or by an emergency unit to the Dermatology and Allergy Center of Hôpital Tenon, Paris, between 2006 and 2009 for evaluation of a history of NSAID and/or APAP (acetaminophen, paracetamol) hypersensitivity were included in the study. Among them, 5 patients had APAP hypersensitivity alone. Half of patients according to the physician report or the patient's declaration had a history of hypersensitivity to one of these drugs during the last year, for the other half it was older.

NSAID-induced or exacerbated urticaria or angiooedema and associated symptoms were clinically classified [21, 28, 29], and severity of clinical symptoms was graded according to the published works [18]. Hypotension was defined as a systolic blood pressure below 100 mm. Patients with recurrent angiooedema were investigated for complement fractions C3 and C4 and C1 inhibitor, but their results were in normal ranges.

Patients suspected of immediate or delayed allergy to an NSAID were discarded. Those with asthma or Widal syndrome were not included because they were followed up in the department of respiratory system diseases.

The study protocol was in accordance with the local ethical committee guidelines, and all subjects gave their consent before being included. Tests were done only for a diagnostic purpose.

### 2.2. Controls

Leukocytes from 12 normal subjects, members of the hospital's staff, and 8 patients with allergic reactions induced by drugs other than NSAIDs were used as controls for BAT, MAT, and NAT. All the controls had never experienced NSAID hypersensitivity and had taken at least once 1 g aspirin within the last 12 months.

### 2.3. Skin Tests

Skin tests (STs), prick ST and intradermal ST, were done as previously described [30, 31].

### 2.4. Oral Challenge Test

In this study no oral challenge test with NSAIDs was done for a diagnostic purpose. In patients needing an analgesic antipyretic drug, double blind, placebo-controlled, orally given APAP tests were carried out when the STs were negative. Protocol for patients needing NSAID therapy was similar, they received selective NSAIDs celecoxib or nimesulide. All the oral challenges were done by the same practitioner.

### 2.5. NSAIDs for Flow Cytometry

They were purchased in solution for intravenous or intramuscular use. Acetyl salicylate lysine (Aspegic, Sanofi-Aventis), acetaminophen (APAP, paracetamol, perfalgan, Bristol-Myers Squibb), ketoprofen (profenid, Sanofi-Aventis), diclofenac (voltaren, Novartis Pharma) and celecoxib (Celebrex, Pfizer Inc.) were diluted in the dilution buffer (2 mM MgCl_2_, 1.2 mM CaCl_2_, and 2 g/L bovine serum albumin in phosphate-buffered saline, PBS). The dilution buffer was used, instead of a NSAID, as the negative control in two tubes, and calcium ionophore 2.5 *μ*g/mL (Sigma) was used as the positive control. Calcium ionophore induced CD63 upregulation in at least 50% of basophils and monocytes and 25% of neutrophils.

### 2.6. Antibodies

R-phycoerythrin-conjugated anti-CD33 monoclonal antibody (MAB), fluorescein-isothiocyanate- (FITC-) conjugated anti-CD63 MAB, R-phycoerythrin-conjugated anti-CD203c MAB, tandem dye R-phycoerythrin-cyanin-5.1-conjugated anti-CD45 MAB, and ECD- conjugated (tandem dye phycoerythrin-Texas red) streptavidin were purchased from Beckman Coulter-Immunotech, Marseille, France. Biotinylated goat anti-human IgE polyclonal antibody was purchased from Vector Laboratories, Burlingame.

### 2.7. Leukocyte Activation Tests

The BASIC (basophils isolated from blood and analysed by flow cytometry) assay was done as previously described [30, 31]. Lithium-heparin anticoagulated peripheral blood was centrifuged at 500 g for 20 minutes at 20°C on a layer of ficoll (*d* = 1.077). The lymphocyte layer and leukocytes suspended in ficoll between lymphocytes and red cells were harvested. After lymphocytes the largest population among these cells were neutrophils, then monocytes, and basophils were the smallest one.

100 *μ*L aliquots of the leukocyte suspension containing 10^6^ leukocytes were mixed with 100 *μ*L of antigen or dilution buffer and then incubated for 30 min at 37°C in a CO_2_ (7%) incubator, after which the cells suspensions were fixed by the addition of 50 *μ*L of 1% paraformaldehyde in PBS.

The leukocytes were then quadruple labelled by adding 10 *μ*L FITC-conjugated anti-CD63 antibody, 20 *μ*L R-phycoerythrin-conjugated anti-CD33 antibody 10 *μ*L tandem dye R-phycoerythrin-cyanin-5.1-conjugated anti-CD45 antibody, and 1/50 diluted 50 *μ*L biotinylated anti-IgE antibody. For the analysis of the CD203c upregulation, basophils were quadruple labelled using the same antibodies, except that the anti-CD33 antibody was replaced by 20 *μ*L R-phycoerythrin-conjugated anti-CD203c. After incubation and washing biotinylated anti-IgE antibodies were labelled by adding 10 *μ*L ECD-conjugated (tandem dye phycoerythrin-Texas red) streptavidin.

**Figure 1:**
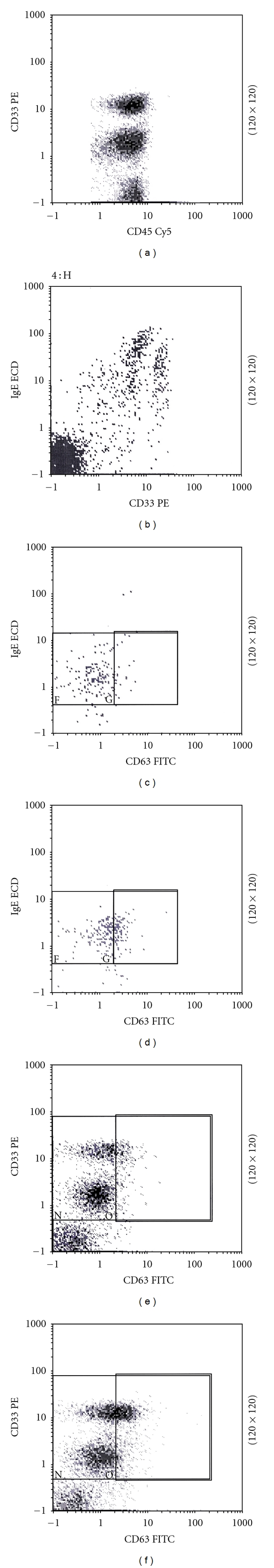
Detection by flow cytometry of CD63 upregulation on leukocytes activated by NSAIDs. (a) and (b) Targeting of leukocytes: (a) shows at the top CD33 bright cells, the monocytes, CD33 dim cells, polymorphonuclears, and CD33-negative cells, lymphocytes. (b) Shows among anti-IgE-labelled cells, CD33dim cells, the basophils, and C33 bright, the monocytes. The percentage of monocytes among IgE-labelled cells varies from 0 to 50%, depending on the patient. (c) and (d) Activation of basophils: (c) basophils incubated with buffer. (d) Basophils incubated with 1 mg/mL of ASA and upregulation of CD63 on 30% of basophils. (e) and (f) Activation of monocytes and neutrophils. (e) Monocytes at the top, and neutrophils under, incubated with buffer. (f) After incubation with 1 mg/mL of ASA, upregulation of CD63 on 25% of monocytes and 5% of neutrophils.

Analysis of leukocytes surface markers was performed on an Epics XL flow cytometer (Beckman Coulter, Marseille, France). On the histogram defined by forward scatter and side scatter, the first gating was done by a bit map around lymphocytes and monocytes, basophils were found in this gate. Another gate was done around polymorphonuclear leukocytes, for neutrophils. The next gatings were done for basophils around IgE+ CD45+ CD33dim cells (Figure 1). These cells previously had been identified as basophils [31–33]. Monocytes were gated around CD45 bright and CD33 bright cells [33], a small proportion of them being IgE+ [31]. The second gating for neutrophils was done around CD45+ CD33dim IgE− cells (Figure 1). When CD203c, a specific marker of basophils [34, 35] was used, basophils were gated around CD203c+ IgE+ CD45+ cells.

In each assay, upregulation of CD63 was measured on at least 400 cells for basophils, monocytes, or neutrophils.

### 2.8. Cut-Off for Positive Results

The tube-to-tube reproducibility was determined by labelling leukocytes of 10 patients and counting 12 tubes per patient. A cut-off value for positive results was chosen at 2 standard deviations (6%) exceeding the value of the nonstimulated control tube.

**Figure 2:**
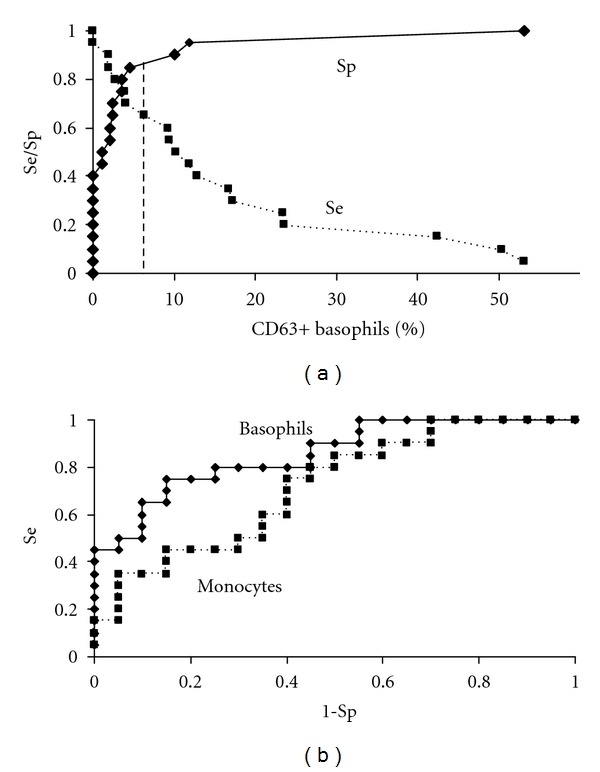
ROC curves for basophils and monocytes incubated with 1 mg/mL of ASA. (a) shows the sensitivity (Se) and the specificity (Sp) of BAT. The cut-off value was determined at 6% of activated basophils (vertical dashed line). (b) shows ROC curves for the activation of basophils and monocytes. The diagnostic performance of the activation of basophils was better (area under the curve for activated basophils 0.855) than that of the activation of monocytes (area under the curve 0.718), *P* < 0.001.

The best cut-off value for activated basophils and monocytes was established by analysing the receiver operating characteristic (ROC) curves of results observed in a selected group of patients with severe hypersensitivity versus controls (Figure 2). The optimal cut-off point deduced from ROC curves was approximately 6% of activated cells. It was similar to that calculated in tube-to-tube reproducibility (see above).

### 2.9. Statistical Analysis

Results of BAT and MAT were blind-analysed, the operator was not informed of the patients' diagnosis. Conversely, the diagnosis of allergy was done before the results of BAT and MAT were available. Statistical analysis was done with *χ* ^2^, paired *χ* ^2^, a two-tailed Fisher's test, Wilcoxon's nonparametric test, and Spearman's rank correlation.

## 3. Results

### 3.1. Clinical History and Classification of Patients with NSAID Hypersensitivity

Among 60 patients with NSAID hypersensitivity, 30 had no underlying disease and had recovered when they abstained from taking NSAIDs. These patients were diagnosed as having NSAIDs-induced urticaria/angioedema according to [21]. The most frequent and characteristic clinical manifestation of the NSAID hypersensitivity was facial angiooedema without flares, observed in 20 patients (67%) (*P* > 0.01).

The remaining 30 patients had chronic and/or episodic (intermittent) idiopathic urticaria and/or angiooedema (C/E IU). NSAID-induced angiooedema was as frequent as urticaria. These patients were diagnosed as having NSAIDs-exacerbated urticaria/angioedema according to [21]. The demography of patients is described in Tables 2 and 3.

Forty-two among 60 patients (70%) had histories of hypersensitivity induced by at least two chemically distinct NSAIDs (Table 2). For the remaining 18 patients we cannot settle if they were true single- or multiple-NSAID reactors because they abstained from taking NSAIDs after the first hypersensitivity reaction. Twenty patients (33%) had also a clinical history of APAP hypersensitivity associated with NSAID hypersensitivity (not shown). All patients with APAP hypersensitivity had a negative ST to APAP. Out of 20 patients with clinical history of APAP and NSAID hypersensitivity and 5 patients with only APAP hypersensitivity, respectively, 5 (25%) and 1 (20%) had APAP oral challenge test positive.

Twenty-two patients with a history of urticaria or angiooedema and with at least one visceral disorder hypotension, laryngeal oedema, dyspnoea, abdominal pain, vomiting or diarrhoea after NSAID intake, were classified as having had a hypersensitivity of grade II (Table 4). After NSAID intake, 21 reacted before 6 h (21/22 = 95%, median 1 h). In contrast, in patients with a history of urticaria or angiooedema with no visceral involvement (grade I), hypersensitivity occurred later (median 8 h) (*P* < 0.0001) (Table 3).

 Among 18 patients with only one known NSAID hypersensitivity, 7 had at least one visceral disorder, frequency (7/18) was similar to patients with hypersensitivity induced by at least two chemically distinct NSAIDs (15/42). No clinical or biological data could discriminate one group from the other.

### 3.2. Tests for the Detection of In Vitro Activated Leukocytes by NSAIDs

Targeting of leukocytes and detection of CD63 upregulation on the membrane of activated cells were done as indicated in Figure 1. A cut-off value for positive results was chosen at 2 standard deviations (in tube-to-tube reproducibility), 6% exceeding the value of the nonstimulated control tube (see Section 2 for more details). This value was similar to the optimal cut-off point deduced from ROC curves for the basophil activation test (BAT) or the monocyte activation test (MAT) (Figure 2).

### 3.3. CD63 Upregulation Induced by ASA

#### 3.3.1. On Basophils

Twenty-two out of 60 patients with a history of NSAID hypersensitivity had a positive BAT to ASA at 1 mg/mL; therefore the sensitivity was 37%. Two controls among 20 (10%) had positive BAT to ASA, the specificity of the test could be estimated close to 90%.

**Figure 3:**
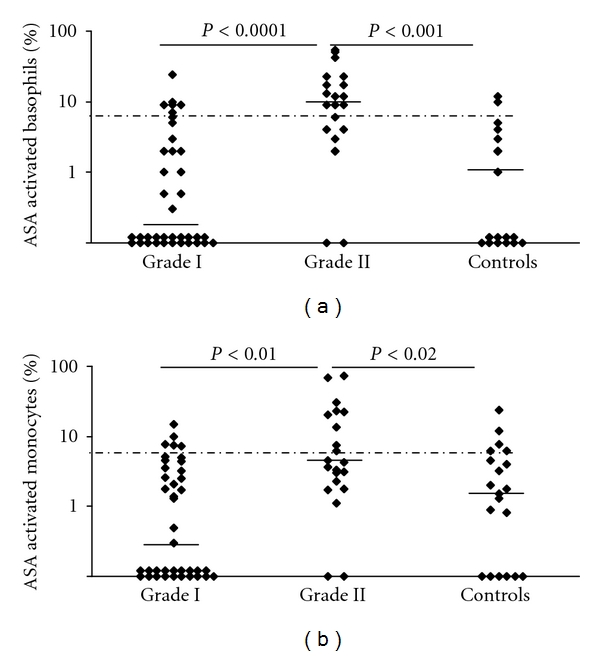
Activation of patients' and controls' basophils and monocytes incubated with 1 mg/mL of ASA. (a) Basophils, (b) monocytes. Grading of reactions in the NSAID hypersensitivity was done according to severity of clinical symptoms [18] (Table 3). The median value is shown by the horizontal bold lines. The cut-off for a positive BAT or MAT was determined at 6% of activated cells (dashed line). Statistical analysis was performed by Wilcoxon's nonparametric test.

In patients suffering from NSAID hypersensitivity restricted to cutaneous reaction (grade I), positive BAT to ASA was not more frequent than in control group (Figure 3 and Table 5).

In contrast, in patients who had had a grade II hypersensitivity, basophils were more strongly activated and BAT was more frequently positive (14/22 = 64%) than in patients with grade I (8/38 = 21%) or controls (Figure 2 and Table 5) (*P* < 0.001, Wilcoxon's nonparametric test). Therefore, a positive BAT in a patient with NSAID hypersensitivity had for a grade II hypersensitivity a positive predictive value of 64% (14/22). The negative predictive value was 79%; only 8 out of 38 patients with a negative BAT had had a grade II hypersensitivity.

A positive BAT to ASA correlates with the precocity of the hypersensitivity. The patients with a positive BAT had reported an interval of 2.5 hours (median value) between NSAID intake and symptoms. On the other hand among patients with negative test the interval was 8 hours (*P* < 0.001).

#### 3.3.2. On Monocytes and Neutrophils

Though less sensitive (46%), results of MAT correlate quite well with BAT (Spearman's rank correlation, *r* = 0.71 for ASA, *r* = 0.49 for diclofenac, *P* < 0.001). The mean percentage of activated monocytes with ASA was greater in patients with grade II hypersensitivity than in those with grade I (*P* < 0.01) or controls (*P* < 0.02, Wilcoxon's nonparametric test) (Figure 2). In control group, in comparison with BAT, the number of results exceeding the cut-off was increased and not significantly different from that of patients. Therefore, the specificity of MAT was relatively low, 75% (Table 5).

Activation of neutrophils with ASA was rather insignificant (Table 4).

### 3.4. Results Observed with Other NSAIDs and APAP

Activation by diclofenac of basophils and monocytes was of the same magnitude of ASA and results correlated rather well (Spearman rank correlation, *r* = 0.59, *P* < 0.01). In contrast, it activated neutrophils better than ASA (Table 5, *P* < 0.01). The mean percentage of activated basophils was greater in grade II patients (median 4.3, range 28–0%) than in grade I patients (median 0.7, range 19–0%) (*P* < 0.03, Wilcoxon's nonparametric test). However, the number of values exceeding the cut-off was not so great to be significant between grade II, grade I or controls (*P* = NS) (Table 4). Ketoprofen, celecoxib, and APAP activated basophils, monocytes, or neutrophils, with values exceeding the cut-off in 10 to 16% of patients and in 0 to 5% of controls (results not shown). APAP did not activate significantly cells of patients with NSAID and APAP hypersensitivity (*n* = 20) or APAP hypersensitivity alone (*n* = 5) (results not shown).

### 3.5. Optimal NSAID Concentrations Activating Leukocytes

Each NSAID and APAP were tested at three ten-fold serial dilutions (Table 1). For each drug the highest concentration was calculated in relation with usual pharmacological doses and tested on leukocytes in order to check the absence of toxic effects. For ASA and diclofenac, available solutions for IV and IM use were at least diluted 1/200 to obtain a final concentration of 1 mg/mL for ASA and 0.125 mg/mL for diclofenac. We avoided 1/20 diluted solutions because they seemed toxic. The percentage of positive tests decreased with increasing dilution; at 1/2000 they were two times less frequent than at 1/200 (results not shown). Results shown are those observed at the dilution 1/200 (Table 4). For ketoprofen and APAP, as they seldom activate leukocytes, available solutions were less diluted: 1/20, 1/200, and 1/2000 (final concentration in Table 1). No toxic effect had been observed with these dilutions.

### 3.6. Comparison of Two Basophil Activation Markers, CD63 and CD203c

In 23 patients and 8 controls, basophils were double labelled with the two activation markers. Activation of patients' basophils with ASA, diclofenac, or ketoprofen was detected 32 times with CD63, 6 times with CD203c, and 4 times with both CD63 and CD203c (results not shown). CD63 was at least 5 times more sensitive than CD203c (*P* < 0.0001). In the control group, basophils activation was detected 5 times with CD63 and 10 times with CD203c. NSAIDs-induced upregulation of CD203c was not significantly different between patients and control group.

## 4. Discussion

The sensitivity (37%) and specificity (90%) of BAT for the diagnosis of NSAID induced hypersensitivity were low. These values rather agree with published results. The sensitivity of BAT was assessed between 33 and 77% with ASA and between 17 and 52% with diclofenac [7, 22, 23, 26, 36–38]. Our cut-off for positive results, at 6% of activated basophils, was slightly higher than the published values. In order to improve the sensitivity of the test, instead of the results observed with a single NSAID, those observed with ASA, diclofenac, and naproxen were combined in an index (ADN index) [26]. As a consequence the sensitivity increased from 43% to 65%.

Our study further shows that, though less frequent than on the basophils, ASA upregulates CD63 on the monocytes of some patients with NSAID hypersensitivity and of some control subjects who tolerate ASA well. Because BAT and MAT have a low sensitivity and specificity in the diagnosis of NSAID hypersensitivity, this raises the question of the clinical significance of a positive test.

Our results suggest a linkage between positive BAT or MAT with ASA and a history of a severe NSAID hypersensitivity. Indeed positive tests were more frequent in patients with severe hypersensitivity than in patients with only cutaneous symptoms or controls (*P* < 0.001). Conversely, in patients with only cutaneous symptoms after NSAID intake the frequency of positive BAT or MAT was quite similar to that in controls. Because the patients with NSAID hypersensitivity are heterogeneous, the conflicting results could be explained by bias of selection [7, 22, 23, 26, 36–38]. Stratification of the patients according to the severity of the clinical symptoms has not been used in the appraisal of the clinical significance of a positive test.

The patients included in this study had been suspected of a nonallergic hypersensitivity to NSAIDs. For most of them this was confirmed because 70% had histories of hypersensitivity with at least two chemically distinct NSAIDs; moreover, in addition to basophils, monocytes were activated in vitro by NSAIDs. Because activation of monocytes is not IgE dependent, a positive MAT might contribute to identify a nonallergic hypersensitivity. Among 18 patients with only one known NSAID hypersensitivity, 7 patients were MAT positive with ASA or diclofenac.

 Results observed in BAT and MAT agree with clinical studies about harmlessness of celecoxib, a selective COX-2 inhibitor [4, 5]. It is a poor activator when compared to ASA or to diclofenac. However, results observed with ketoprofen are contradictory: it is one of the strongest COX-1 inhibitors [15, 16] (Table 1) but was a poor activator in BAT and MAT even tested at higher concentrations than ASA or diclofenac. This suggests that inhibition of COX-1 is not enough to activate leukocytes. It remains to determine the signification of cellular responses to NSAIDs and the correlation with the disease evolution. Though nonimmune, NSAID hypersensitivity in most patients is acquired and occurs around the age of forty years (Table 2). In a follow up for 4 years, a third of the patients recovered [3]. Intolerance to NSAID might precede by years the onset of CIU [39].

Twenty out of 60 (33%) patients reported in addition to NSAID APAP hypersensitivity, but, when tested with APAP, skin tests, BAT, and MAT were negative. The mechanism by which APAP affects fever and pain is still debated. It remains a weak COX inhibitor, but it is more potent in inhibiting COX-2 than COX-1, like selective COX-2 inhibitors [17].

One team published contradictory results about the sensitivity of the BAT, it reported that diclofenac induces basophil degranulation without increasing CD63 expression in sensitive patients [25]. However, the highest concentration of diclofenac they used was 10 *μ*g/mL, which is 8 times lower than the concentration used in the other published works [22, 23, 26]. We observed, in agreement with published works for basophils and also for monocytes, that they were better activated by increasing the concentration of NSAIDs. But the too high concentrations (≥5 mg/mL for ASA, ≥1.25 mg/mL for diclofenac) were toxic or activated nonspecifically the basophils of the controls [36, 37, 40].

CD203c upregulation, compared to CD63, poorly detected basophil activation by NSAIDs, but there is a controversy about CD203c upregulation by NSAIDs [37, 38]. Discrepancies about the sensitivity of CD203c compared to that of CD63 might have different explanations [31, 34, 41].

In summary, in patients suffering from NSAID hypersensitivity restricted to cutaneous reaction, in vitro activation of basophils or monocytes by NSAIDs was similar to that of control subjects. In contrast, a group of patients who had had early and quite severe reactions (grade II) had with ASA significantly stronger activation of basophils and monocytes.

## Figures and Tables

**Table 1:** Comparison of NSAIDs and acetaminophen concentrations incubated with leukocytes to serum concentrations at usual therapeutic dosage and to 50% inhibitory concentrations (IC50) of cyclooxygenase-1 and -2.

Drug	Concentrations				
	Tested*		In serum**	IC50***	
	mg/mL	*μ*M	*μ*M	COX-1	COX-2
ASA	0.01–0.1–1	31–310–3100	111	4.45	13.88
Diclofenac	0.0013–0.013–0.13	4.2–42.2–422	6.1	0.26	0.01
Ketoprofen	0.025–0.25–2.5	98.3–983–9830	9.4	0.11	0.88
Celecoxib	0.005–0.05–0.5	13.1–131–1310	1.6	82	6.8
Acetaminophen	0.01–0.1–1	66.1–661–6610	117	113.7	25.8

*Each NSAID and and acetaminophen (APAP) were tested at three ten-fold serial dilutions.

**Serum concentrations at usual therapeutic dosage.

***Concentration of drug that inhibited 50% of COX-1 in platelets or COX-2 in monocytes [15–17].

**Table 2:** Description of patients suffering from NSAID hypersensitivity.

Group of patients*		Age	Sex ratio	Atopy**	C/E IU***	HS ≥ 2**** NSAIDs
Grade I	*n* = 38	44	29F/9M	9 (24%)	18 (47%)	25 (66%)
Grade II	*n* = 22	43	16F/6M	7 (32%)	12 (55%)	17 (77%)

Total	*n* = 60	44	45F/15M	16 (27%)	30 (50%)	42 (70%)

*Patients with a history of urticaria or angiooedema and no visceral disorder were classified as having had a hypersensitivity of grade I and those with at least one visceral disorder were classified as grade II according to published works [18].

**Patients had history of atopic dermatitis, allergic rhinitis, or asthma, but they had healed at the time of NSAID hypersensitivity.

***The patients with “C/E IU” had chronic or episodic idiopathic urticaria or angiooedema; NSAID hypersensitivity was diagnosed when symptoms worsen relapsed, or were unusual and recovered when they stopped NSAID intake.

****Patients who had histories of hypersensitivity induced by at least two chemically distinct NSAIDs.

**Table 3:** Skin symptoms and time to onset of the NSAID-induced hypersensitivity.

Group of patients		Time to onset*		NSAID-induced skin symptoms		
		Median	<6 H	AO	AO + Urticaria	Urticaria
Grade I	*n* = 38	8 H	14 (37%)	18 (47%)	9 (24%)	11 (29%)
Grade II	*n* = 22	1 H	21 (95%)	12 (55%)	7 (32%)	3 (14%)

Total	*n* = 60	4.5 H	35 (58%)	30 (50%)	16 (27%)	14 (23%)

*The time to onset of symptoms after NSAID intake was shorter in patients with grade II hypersensitivity than in patients with grade I (*P* < 0.0001).

**Table 4:** Description of severe reactions (grade II) observed in patients with NSAID hypersensitivity.

Grade II reactions/time to onset*		Visceral disorders (VD)				
		Laryngeal oedema	Dyspnoea**	Hypotension	G-intestinal disorders	At least one VD***
<6 H	*n* = 21	9 (43%)	8 (38%)	6 (29%)	3 (14%)	21 (100%)
8 H	*n* = 1	1	0	0	0	1

Total	*n* = 22	10 (45%)	8 (37%)	6 (27%)	3 (14%)	22 (100%)

*Time to onset of symptoms after NSAID intake. **Dyspnoea observed in patients with no laryngeal oedema. ***Patients with a history of urticaria or angiooedema and with at least one visceral disorder were classified as having had a hypersensitivity of grade II according to published works [18].

**Table 5:** Activation of basophils (BAT), monocytes (MAT), and neutrophils (NAT) induced in vitro by ASA (1 mg/mL) or diclofenac (0.125 mg/mL).

		Patients with positive tests*					
Group of patients		BAT+		MAT+		NAT+	
		ASA	Diclofenac	ASA	Diclofenac	ASA	Diclofenac
NSAID HS	*n* = 60	22 (37%)	20 (33%)	14 (23%)	15 (25%)	8 (13%)	16 (27%)
Grade I**	*n* = 38	8 (21%)	10 (26%)	4 (11%)	9 (24%)	6 (16%)	12 (31%)
Grade II	*n* = 22	14 (64%)	10 (46%)	10 (46%)	6 (27%)	2 (9%)	4 (19%)
Controls	*n* = 20	2 (10%)	5 (25%)	5 (25%)	3 (15%)	0 (0%)	2 (10%)

*The optimal cut-off point for a positive test deduced from ROC curves was 6% of activated cells (see Section 3). Activation was detected by CD63 upregulation. **Grading of reactions according to severity of clinical symptoms (Tables 2 and 4).
